# Workplace-based assessments of entrustable professional activities in a psychiatry core clerkship: an observational study

**DOI:** 10.1186/s12909-021-02637-4

**Published:** 2021-04-21

**Authors:** Severin Pinilla, Alexandra Kyrou, Stefan Klöppel, Werner Strik, Christoph Nissen, Sören Huwendiek

**Affiliations:** 1 University Hospital of Old Age Psychiatry and Psychotherapy, University of Bern, Bern, Switzerland; 2 Department for Assessment and Evaluation, Institute for Medical Education, University of Bern, Bern, Switzerland; 3 University Hospital of Psychiatry and Psychotherapy, University of Bern, Bern, Switzerland

**Keywords:** Entrustable professional activities, Entrustment, Workplace-based assessment, Undergraduate medical education, Clerkship

## Abstract

**Background:**

Entrustable professional activities (EPAs) in competency-based, undergraduate medical education (UME) have led to new formative workplace-based assessments (WBA) using entrustment-supervision scales in clerkships. We conducted an observational, prospective cohort study to explore the usefulness of a WBA designed to assess core EPAs in a psychiatry clerkship.

**Methods:**

We analyzed changes in self-entrustment ratings of students and the supervisors’ ratings per EPA. Timing and frequencies of learner-initiated WBAs based on a prospective entrustment-supervision scale and resultant narrative feedback were analyzed quantitatively and qualitatively. Predictors for indirect supervision levels were explored via regression analysis, and narrative feedback was coded using thematic content analysis. Students evaluated the WBA after each clerkship rotation.

**Results:**

EPA 1 (“Take a patient’s history”), EPA 2 (“Assess physical & mental status”) and EPA 8 (“Document & present a clinical encounter”) were most frequently used for learner-initiated WBAs throughout the clerkship rotations in a sample of 83 students. Clinical residents signed off on the majority of the WBAs (71%). EPAs 1, 2, and 8 showed the largest increases in self-entrustment and received most of the indirect supervision level ratings. We found a moderate, positive correlation between self-entrusted supervision levels at the end of the clerkship and the number of documented entrustment-supervision ratings per EPA (*p* < 0.0001). The number of entrustment ratings explained 6.5% of the variance in the supervisors’ ratings for EPA 1. Narrative feedback was documented for 79% (*n* = 214) of the WBAs. Most narratives addressed the Medical Expert role (77%, *n* = 208) and used reinforcement (59%, *n* = 161) as a feedback strategy. Students perceived the feedback as beneficial.

**Conclusions:**

Using formative WBAs with an entrustment-supervision scale and prompts for written feedback facilitated targeted, high-quality feedback and effectively supported students’ development toward self-entrusted, indirect supervision levels.

**Supplementary Information:**

The online version contains supplementary material available at 10.1186/s12909-021-02637-4.

## Background

Introducing entrustable professional activities (EPAs) to competency-based undergraduate medical education (UME) has led to new approaches for the design of workplace-based assessments (WBAs). EPAs are observable clinical tasks and serve as units of assessment that are often based on entrustment-supervision scales [1–6]. WBAs (i.e., any type of structured assessment done in the workplace such as the Mini-clinical exercise or clinical work sampling) serve multiple purposes [7, 8]. In a low-stakes context, they are intended to create opportunities for structured observation, feedback, and to support the achievement of competency-based learning goals (assessments for learning) [7, 9–11]. In the context of linking clinical UME and graduate medical education (GME) curricula [12, 13]— and short clerkship rotations—the emphasis should be on maximizing the value of formative WBAs, as described in GME [14]. This value depends on the context, content, and quality of the feedback resulting from the WBAs [15–21]. While a number of studies have explored the potential of WBAs for generating high-quality narrative feedback in GME [22–25], little is known about the relationship between WBAs based on an entrustment-scale, their narrative feedback output, and the perceived need for supervision (i.e., self-entrustment) in early-stage clinical students. In particular, changes in self-entrustment can be used as an indicator of self-efficacy [26–29]. Thus, developing higher levels of self-entrustment is relevant for self-regulated learning in clinical workplaces.

WBAs have become a central part of many graduate training programs [20, 30–33] and are increasingly used to assess EPAs and competencies in undergraduate clinical training programs as well [2, 4, 34–37]. Typically, they are used to support the direct or indirect observation of trainees’ clinical activities and to provide assessment information for both low- and high-stakes purposes. Despite the potential of WBAs to provide formative feedback and their key role within assessment programs [8, 11], major feasibility issues have been identified in workplaces. These include a lack of understanding regarding the purpose of WBAs from both trainers and trainees, time constraints, and a lack of training within faculties [21, 32]. Duijn et al. [21] identified specific criteria for meaningful feedback taken from students’ perspectives on EPAs. These corresponded to the general feedback quality criteria described by Lefroy et al. [19], which included reinforcement, key point identification, strategy development, and whether feedback is actionable.

The requirements and complexity involved in aligning valid learning goals such as EPAs, rating scales, and feedback narratives from WBAs has been explored in GME [22, 38] and UME [21, 34, 39]. However, we are unaware of any studies that have addressed the content and quality of narrative feedback resulting from WBAs based on entrustment-supervision scales in psychiatry clerkships. Prospective entrustment-supervision scales differ from traditionally abstract WBA scores in that each supervision level directly reflects the degree of supervision required when subsequently performing a clinical activity [4, 6, 40]. The level of supervision in ad hoc entrustment decisions (i.e., an instant entrustment decision in a real working context) is set by a clinical supervisor.

In contrast, self-entrustment (that is, evaluating one’s ability to perform a clinical activity under a given level of supervision) [27], as related to self-efficacy, is a students’ judgment concerning the ability to face a potentially challenging situation [41]. Hence, higher self-entrustment has been identified as an important factor for the active engagement of students with clinical work and self-regulated learning [27, 41]. Due to a number of different (self-) entrustment-supervision scales in use [4, 35, 42], the relationship between self-entrustment and formative WBAs with documented ad hoc entrustment ratings and narrative feedback remains unclear. A further criticism of learner-initiated WBAs is that medical students may selectively pick favorable clinical activities for graded WBAs, resulting in selection bias [43]. To our knowledge, the question of how clerkship students use a mandatory, but purely formative and self-initiated, WBA with entrustment-supervision scales has not been studied in a core clerkship setting.

Therefore, we conducted an observational study to explore the usefulness of a developmental (that is formative) assessment tool (a structured, paper-based observation format) designed to assess Swiss core EPAs in a psychiatry clerkship based on a prospective supervision-entrustment scale. Formative assessment in our study included aspects that supported learning in a clinical workplace (assessment *for* learning) in contrast to summative assessments *of* learning. Our primary outcome parameter was the change in students’ self-entrusted supervision level per core EPA following a core clerkship rotation in psychiatry. Secondary outcome parameters were the timing and frequencies of learner-initiated WBAs, potential predictors for reaching self-entrusted indirect supervision levels per core EPA, the frequency, quality and content of narrative feedback resulting from this formative assessment tool, and students’ evaluations of the WBA tool.

## Methods

### Study design

We chose an observational, prospective cohort study design.

### Context and participants

Students were prepared for their core clerkship rotations (in the 4th year of medical school) with didactic lectures organized by specialty; clinical-skills training for taking patient histories as well as physical and mental examinations; and communication training with standardized patients. During the core clerkship year, students rotated through nine different specialties, lasting two to four weeks per clerkship rotation, in teaching hospitals affiliated with the University of Bern. The majority of students remained on the same ward as members of the clinical team during their four-week psychiatry rotation. Clinical supervision, which often including the signing-off on WBAs, was typically provided by residents, psychologists, and attendings (see Table 1). A national competency-based learning catalogue was introduced for the core clerkship year in UME in 2019 at the University of Bern, Switzerland [44]. The Principal Relevant Objectives and Framework for Integrative Learning and Education in Switzerland (PROFILES) was based on the CanMEDS roles and nine core EPAs (see Table 2 for descriptions) [44]. The CanMEDS roles have been extensively described elsewhere [44, 45]. We slightly modified the core EPA titles (e.g., instead of “Take a patient’s history” we used “Take a patient’s psychiatric history”, or instead of “Contribute to a culture of safety” we used “Identify and report opportunities to improve patient safety in a psychiatric hospital”) to our clinical context.

**Table 1:** Characteristics of the study participants

**Characteristics of the clerkship students**	
Sample	n = 83 (100% of students rotating through our teaching hospital during the study period, representing 33% of the full 2019 clerkship cohort at our medical school)
Age	Average 23.9 (range: 21–34)
**Gender**	
Female	59% (*n* = 49)
Male	41% (*n* = 34)
**Canton of origin**	
	Bern (53%, n = 44), German speaking
	Aargau (12%, *n* = 10), German speaking
	Fribourg (6%, *n* = 5), French and German speaking
	Other (29%, *n* = 24), including Italian speaking
Interest in specialty*	
Before clerkship	10% (n = 8)
After clerkship	17% (*n* = 14)
Overall satisfaction** with clerkship	
Average	4.4 (range: 1–5)
**Number of different raters per student for WBAs during clerkship rotation**	
Average	1.8 (range: 1–4)
One rater	39% (*n* = 32)
Two raters	42% (*n* = 35)
Three raters	17% (n = 14)
Four raters	2% (n = 2)
**Characteristics of the clinical raters (signing-off on WBAs)**	
Total	*n* = 66***
**Gender**	
Female	48% (n = 24)
Male	52% (*n* = 26)
**Health profession group**	
Resident	71% (*n* = 46)
Psychologist	15% (n = 10)
Nurse	5% (n = 3)
Attending	5% (n = 3)
**Place of undergraduate medical education**	
University of Bern	24% (*n* = 12)
Other	76% (*n* = 38)

WBA: workplace-based assessments.

* Measured using a 5-point Likert scale (interest defined as a student marking 4 or 5 agreement on Likert-item “I definitely plan further training in psychiatry”)

** Measured by a 5-point Likert scale (1 = unsatisfied, 5 = highly satisfied)

*** Fifteen raters were not fully categorized due to missing information

**Table 2:**
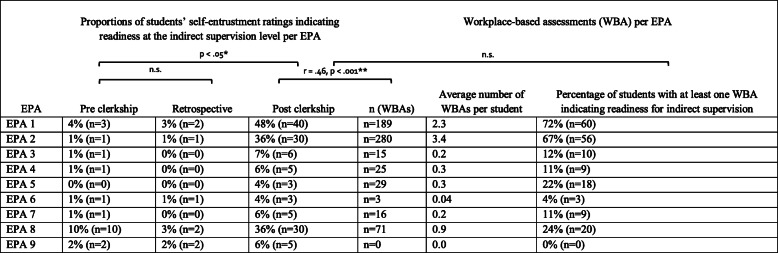
Self-entrustment ratings and workplace-based assessments (WBAs) per entrustable professional activity (EPA)

* Statistically significant at the 0.05-level. ** Statistically significant at the 0.01-level. The Wilcoxon Signed-Rank test was used for self-entrustment rating comparisons, Spearman’s rho for the correlation between the number of WBAs and the post clerkship rating, and the Mann-Whitney U test for a comparison of proportions of post clerkship self-entrustment ratings of students and WBA ratings per EPA (*n* = 9) at the indirect supervision level, total n (students) =83, total n WBAs =628

EPAs based on Principal Relevant Objectives and Framework for Integrative Learning and Education in Switzerland (PROFILES), http://www.profilesmed.ch/

EPA 1: Take a patient’s history, EPA 2: Assess physical and mental status, EPA 3: Prioritize a differential diagnosis, EPA 4: Order and interpret tests,EPA 5: Perform general procedures, EPA 6: Recognize and treat an emergency, EPA 7: Prescribe and develop a management plan, EPA 8: Document and present a clinical encounter, EPA 9: Contribute to a culture of safety

Pre clerkship: Students’ self-entrustment rating on the first day of their clerkship rotation (6-point entrustment scale from 1 = I can only observe this activity to 6 = I can do

this if I can ask for help when needed (indirect supervision))

Retrospective: Retrospective students’ self-entrustment rating on the last day of their clerkship rotation (6-point entrustment scale from 1 = On my first day of the clerkship rotation I could only observe this activity to 6 = On the first day of my clerkship rotation I was able to do this activity if I could ask for help when needed

(indirect supervision))

Post clerkship: Students’ self-entrustment rating on the last day of their clerkship rotation (6-point entrustment scale from 1 = I can only observe this activity to 6 = I can do this if I can ask for help when needed (indirect supervision))

Clerkship students were required to submit at least four documented entrustment-supervision ratings on one or several WBA forms per rotation, of which one had to be for EPA 1 (“Taking a patient’s psychiatric history”) and one for EPA 2 (“Assess physical & mental status). It was the students’ responsibility to collect these WBAs during their clerkship rotations. Clinical supervisors at our teaching hospital have been working with WBAs in clerkship rotations since 2010 and are, therefore, familiar with the general WBA format [34]. Furthermore, clerkship directors attended a two-hour seminar prior to starting the clerkship year on the nature and purpose of the novel WBA tool for EPAs. Supervising residents at our teaching hospital were also instructed during departmental meetings and via email. All assessments were formative, mandatory, and students did not receive any grades. The WBAs did not include the self-entrustment ratings of the students. To successfully complete the clerkship rotation, students needed to hand in all assessments signed by their supervisor.

To support the observation and assessment of EPAs, a form listing the nine adapted core EPAs, together with an entrustment-supervision rating scale, was developed (Fig. S1). The scale was adapted to our context from a published, prospective entrustment-supervision scale for UME [1]. Our scale included six levels of supervision, ranging from “Observe only” (level 1, minimal expectation for students entering the clerkship year) to “The student can do this if he/she can ask for help when needed” (level 6, target level for graduation from medical school), as the highest level allowed at our institution (Fig. S1). Clinical supervisors could rate the supervision level for one or more observed EPAs per form. We also included dedicated space on the assessment form for narrative feedback. To prompt specific and actionable feedback, we added guiding questions (“What was done well?”, “What can be improved?”, “Next steps?”) on the WBA form.

We collected demographic, assessment, and evaluation data from the clerkship students (*n* = 83) during their psychiatry rotation at our academic teaching hospital between March and November 2019. We analyzed the self-entrusted levels of supervision per student per core EPA (see Table 2) at different time points, one on the first day and two on the last day of the clerkship (current level and retrospective level for the first day). This was done to explore changes after the one-month clerkship rotation and retrospective adjustments of the perceived need for supervision.

### Data analysis

All WBAs with supervisors’ ratings were inputted into Microsoft Excel, version 16.37, for analysis. For descriptive analyses per EPA, we included the proportion of students that received an indirect supervision score (level 6 on the entrustment scale) and comparisons of post self-entrustment ratings with supervisors’ entrustment ratings. We ran a Wilcoxon Signed-Rank test for students’ self-entrustment comparisons (pre and post clerkship ratings), a Spearman’s rho correlation to determine the relationship between the post clerkship self-assessed need for supervision per EPA and the number of WBAs per EPA, and a Mann-Whitney U test for a comparison of the proportion of students’ self-entrustment ratings (post clerkship) and the proportion of WBA ratings per EPA at the indirect supervision level. A stepwise regression analysis using SPSS (version 25; IBM Corp, Armonk, NY) was used to identify predictors of WBA entrustment ratings and self-entrustment ratings at the end of the clerkship rotation.

The feedback narratives from all of the WBAs were imported into the qualitative research software MAXQDA (version 20.0.8; VERBI GmbH, Berlin, Germany) for thematic content analysis [46]. We used a published coding framework for the CanMEDS roles [22] to code individual narrative feedback. No further codes emerged from our data analysis. In addition, we defined codes for high-quality feedback based on published guidelines with strong evidence for feedback effectiveness in clinical education [19] (i.e., reinforcement, key point identification, strategy development, self-awareness, EPA-specific, actionable). We added one code for narrative feedback that explicitly stated entrustment. The first ten feedback narratives were fully and independently coded by two researchers. Discrepancies were resolved through discussion, and anchoring examples were used for further coding. One researcher coded the remainder of the full data set. Samples from the full data set were used to regularly check that there were no new coding discrepancies. All of the students from all of the teaching hospitals in psychiatry were invited to evaluate the novel WBAs after each clerkship. The other psychiatry teaching hospitals used a general WBA form for all core clerkships (and not adjusted to the psychiatry clinical context), which was also based on a prospective entrustment-supervision scale and prompts for narrative feedback.

Since these evaluations were conducted externally, the data included students from the pilot month and were only available in a summary report. We compared the students’ WBA evaluations with the previous year (if data were available) where Mini Clinical Evaluation Exercises (Mini-CEX) were used as the WBA format [34].

### Ethics

The ethics committee of the canton of Bern reviewed the research design and exempted this study from additional ethical approval. Confidentiality and anonymity regarding electronic data was maintained throughout the study. Any names or potentially identifying information were removed before analyzing the data. All direct quotes were translated from German to English.

## Results

Clerkship students in our sample (*n* = 83) submitted 628 distinct entrustment-supervision ratings from clinical supervisors (each rating corresponded to one EPA) on 271 WBA forms containing one or more ratings. There was an average of three forms per student and two ratings per form. The average number of observed and documented EPAs per student was 7.5 (SD = 1.2), which exceeded the minimum requirement of four per student. In addition, each student filled out three self-entrustment ratings per core EPA (Table 2). Except for the pilot data from the first month, the full clerkship-year data (nine months) were included in the final analysis.

### Participant characteristics

Students in our sample represented one third of the 2019 clerkship cohort and 53% originated from the canton of Bern. The remainder of the students originated from other cantons in Switzerland, including three different language regions. Females were marginally over-represented in the sample (female-to-male ratio = 3:2), and the average student age was 24 years. Interest in psychiatry as a specialty was low at the beginning of the clerkship rotation, with only 10% planning further training. This increased to 17% after the rotation.

A total of 66 different raters signed-off on the WBA forms during the study period. Most students (81%) had their WBAs signed-off by one or two different raters (Table 2). The gender ratio of the raters was equal. The majority of WBAs were signed-off by clinical residents (71%), followed by psychologists (15%), nurses (5%), and attending physicians (5%). The majority of resident raters (76%) had undertaken their undergraduate medical training at a different medical school. Descriptive statistics of the study participants are summarized in Table 1.

### Change in self-entrustment ratings and clinical supervisors’ ratings per EPA

With regard to self-entrustment ratings, 87% (*n* = 72) of the students did not perceive themselves as ready for indirect supervision for any of the nine EPAs on the first day of their clerkship rotation. Only a small proportion of students self-entrusted at the indirect supervision level in the beginning of the clerkship rotation, which ranged from 0% (EPA 5) to 10% (EPA 8). Retrospective self-entrustment ratings showed a similar distribution per EPA, except for EPA 8 (“Document & present a clinical encounter”) for which more students felt ready for indirect supervision in the beginning of the clerkship compared to retrospectively (10% versus 2%). The proportion of students that self-assessed as ready for indirect supervision was higher for all of the EPAs at the end of the clerkship (with an average increase of 15% per EPA, range: 3–44%) and showed a similar distribution per EPA compared to supervisors’ entrustment ratings on the WBAs. The overall increase in self-entrusted indirect supervision level was highest for EPAs 1, 2 and 8 (increases of 44, 35 and 26% of students, respectively). Most students received at least one WBA entrustment rating indicating readiness for indirect supervision (level 6 on the supervision scale, Fig. S1) for EPA 1 (72%) and EPA 2 (67%). For EPAs 3–8, the percentage of students receiving at least one indirect supervision rating ranged between 4 and 24% (Table 2). Supervisors rated a higher proportion of students as ready at the indirect supervision level for EPAs 1, 2, 3, 4, 5, and 7 and a similar or smaller proportion for EPAs 6, 8 and 9 in comparison to the end-of-clerkship-self-entrustment ratings of students. We found a moderate, positive, monotonic correlation between the perceived need for supervision-level and the quantity of WBAs per EPA (Spearman rho = 0.46, *n* = 684, *p* < 0.0001). Thus, more WBAs were associated with a higher self-entrusted supervision level (i.e., more independence).

### Predictors for achieving indirect supervision levels per EPA

A regression analysis showed that the month of clerkship rotation, age, gender, quantity of entrustment-supervision ratings, or self-assessed need for supervision at the beginning of the clerkship did not predict the level of self-assessed need for supervision per EPA at the end of the clerkship. However, the number of entrustment ratings explained 6.5% of the variance in the supervisors’ ratings for EPA 1 (F(1,63) =4.408, *p* < 0.05), with an R^2^ of 0.065. For EPA 2, interest in the specialty explained 7.5% of the variance in supervisors’ ratings (F(1,63) =5.103, *p* < 0.05) with an R^2^ of 0.075, and the number of ratings correlated positively with the level of ratings (r = 0.25, p < 0.05). We found no predictors for supervisors’ ratings for EPA 8. The interest in the specialty explained 24% of the variance in the change in perceived need of supervision for EPA 1 (F(1,59) =18.753, *p* < 0.05) with an R^2^ of 0.241 and 13% for EPA 2 (F(1,59) =8.715, p < 0.05) with an R^2^ of 0.129. Interest in the specialty negatively correlated with a change in perceived need of supervision, indicating greater increases of perceived independence for students who were less interested in the specialty (Pearson r = − 0.491, *p* < 0.001 for EPA 1 and r = − 0.359; *p* < 0.01 for EPA 2). For all other EPAs, we could not analyze predictors because too few WBAs were done.

### Timing and frequencies of learner-initiated WBAs

**Fig. 1:**
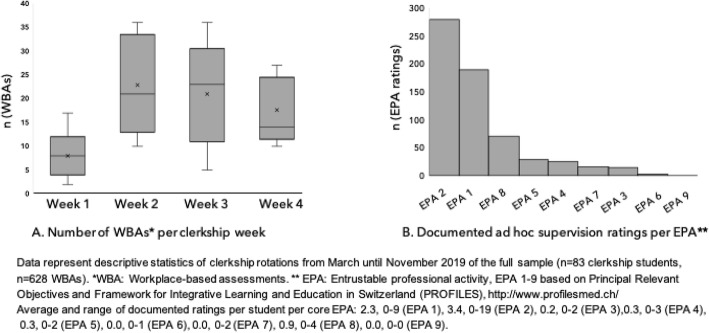
Use pattern of formative workplace-based assessments (WBAs) in clerkship rotations

The average student collected one entrustment-supervision rating in the first clerkship week and two per week during the remainder of the clerkship rotation (Fig. 1a). Most entrustment-supervision ratings were collected for EPA 1 (“Take a patient’s psychiatric history”, *n* = 189), EPA 2 (“Assess physical & mental status”, *n* = 280) and EPA 8 (“Document & present a clinical encounter”, *n* = 71). Except for EPA 9 (“Contribute to a culture of safety”), all other EPAs were used for WBAs, but less frequently (Fig. 1b).

### Narrative feedback resulting from the WBA tool

Students received narrative feedback on 79% (*n* = 214) of all WBA forms (Table 3). On two WBA forms with no narrative feedback we found statements indicating that students had received oral feedback. The average length of the feedback narratives was 12.3 words (SD = 6.1). In terms of content, most narratives addressed the Medical Expert role (77%, *n* = 208), followed by the Professional (17%, *n* = 44) and Communicator roles (15%, *n* = 37). The application of knowledge for structuring a clinical interview, taking a patient history, examining mental status, and performing a physical exam were most frequently mentioned (46%), followed by communication skills with patients and showing compassion and empathy 25% (Table 3). The following quotes illustrate the narrative feedback extracted from the WBA forms. The first one was signed off by a resident, who used reinforcement in their feedback, which relates back to a previous clinical observation. It provides EPA-specific feedback with regard to the mental status exam and communication tactics:

**Table 3:** Narrative feedback on all submitted workplace-based assessment (WBA) forms during psychiatry clerkship rotations between March and November 2019

Content and quality	Applied codes^a^	Proportion of WBA forms (*n* = 271)
Medical Expert^b^		
	Application of knowledge (clinical skills/clinical decision making)	46% (*n* = 125)
	Communication, compassion	25% (*n* = 69)
	Knowledge base	5% (*n* = 13)
	Clinical care and safety	0.4% (n = 1)
Communicator		
	Written	13% (n = 35)
	Oral	2% (*n* = 5)
Professional		
	Work ethic	7% (n = 18)
	Conduct	6% (*n* = 15)
	Work skills	4% (n = 11)
Collaborator		
	Team player	1% (n = 3)
Not related to CanMEDS roles		
	Entrustment	8% (n = 21)
	On-rotation improvement	3% (n = 7)
	Attitude	1% (n = 4)
	Norm reference	0.4% (n = 1)
Quality indicators of feedback		
	Reinforcement	59% (n = 161)
	EPA-specific	42% (n = 113)
	Actionable	32% (n = 87)
	Key point identification	32% (*n* = 86)
	Strategy development	16% (*n* = 42)
	Self-awareness	8% (*n* = 23)

^a^Codes derived from sources described in the methods section

^b^Corresponding CanMEDS roles as referenced in Principal Relevant Objectives and Framework for Integrative Learning and Education in Switzerland (PROFILES), http://www.profilesmed.ch/. We found no feedback narratives corresponding to the roles of Leader/Manager, Health Advocate, or Scholar

“Great structure of clinical interview despite challenging situation, improved focus compared to admission of first patient. Important information [suicidality marked on WBA form] must be addressed directly; sometimes you need to insist a little bit!”

(WBA form 176: EPAs 1 and 2, signed-off by resident. Supervision scale: level 5 for both)

Another salient quote by a supervising nurse illustrates reinforcement and key aspect identification in the context of performing a venipuncture:

“Correct execution [of venipuncture]. Great instruction for patient. [Clerkship student] shows friendly and empathetic interaction with patient. Needs more routine, otherwise well performed. Don’t forget hand disinfection!”

(WBA form 204: EPA 5, signed-off by nurse. Supervision scale: level 2)

Only 8% of the WBAs contained narrative elements that explicitly commented on entrustment (*n* = 21) such as “Independent patient admission, including mental status and documentation” (WBA form 153). In terms of feedback quality, we found different frequencies of high-quality feedback indicators. The most frequent feedback strategy was reinforcement (59%, *n* = 161), followed by specifically commenting on observed EPAs (42%, *n* = 113), and providing actionable feedback (32%, *n* = 87). Some clinical supervisors structured their narratives using the headings “positive” and “negative”. EPAs 1, 2 and 8 were the most frequently addressed in the narrative feedbacks.

### Student evaluations of the WBA tool

All students agreed that the required number of WBAs had been documented (agreement rate at our institution was 100%). With regard to receiving verbal feedback for clinical competencies after WBAs, 92% of students stated that they always or mostly received verbal feedback, and 8% answered that they rarely or never received verbal feedback.

Concerning narrative feedback, 75% of all students stated that they always or mostly received narrative feedback, while 25% said they rarely or never received narrative feedback. On a 6-point Likert scale (1 = I completely disagree, 6 = I fully agree), the overall, average rating for “I benefited from the feedback after workplace-based assessments” was 4.8 (4.4 in the previous clerkship year) and for “The learning goals defined after the workplace-based assessments were actionable” it was 5.1 (4.6 in the previous clerkship year).

## Discussion

The aim of our study was to explore changes in students’ self-entrusted supervision level per Swiss core EPA after introducing a novel WBA format based on a prospective entrustment-supervision scale during a core clerkship rotation in psychiatry. Our results suggest that self-entrustment ratings changed per core EPA over the course of a clerkship rotation. The use of the novel WBA format correlated with an increase in self-entrustment per core EPA. That is, when used in a clerkship rotation, this WBA format was associated with progress towards higher levels of self-entrustment. Students predominantly chose three core EPAs (“Taking a patient’s history”, “Assess the physical & mental status”, and “Document & present a clinical encounter”) for WBAs. The narrative feedback generated with this WBA format centered on aspects of the CanMEDS roles: Medical Expert, Professional, and Communicator. We also found that clerkship students were entrusted and observed in clinical activities by different health professionals on the ward team. Our findings could inform future reforms of national EPA-based frameworks and competency-based curriculum designs in psychiatry for UME.

### Self-entrustment ratings and predictors of achieving indirect supervision levels

Changes in self-entrustment ratings showed a similar distribution pattern as the WBAs across EPAs and a moderate, yet significant, correlation of self-entrustment level and number of WBAs. The number of observed and documented ratings per EPA could explain some of the variance in self-entrustment ratings at the end of the clerkship. The strength of the correlation was comparable to findings from other types of WBAs [15]. Differences in the nature of assessed clinical activities (e.g., taking a history from a psychotic patient versus doing a motivational interview) and complexity levels (e.g., a mental status assessment of a patient with mild depressive symptoms versus an acutely suicidal patient) could have moderated the true association between the number of WBAs and the perceived need for supervision [6, 9]. Clinician educators in UME could use these types of formative WBAs to help students achieve the next supervision level. In our study, students who were initially less interested in the specialty appeared to benefit more from these WBAs in terms of an increase in self-entrustment. This finding provides evidence for the potential of these WBAs to be used as effective assessment tools for supporting self-efficacy-related learning in workplaces, as has been described in the GME context [47].

An important finding of this study was that less than half of the students felt prepared for indirect supervision in any of the core EPAs at the end of their clerkship. As we found no significant retrospective adaption of self-entrustment ratings per EPA, this type of self-assessment appears to be more stable compared to self-assessments of competence [27, 48]. Reflecting on the ability of performing a concrete clinical task might be different than reflecting on a more abstract concept of competence and be less prone to social desirability bias [41]. Taking into consideration that, for most students at our institution, this was the only clinical exposure in psychiatry, it is questionable whether setting a target of an indirect supervision level across all core EPAs is realistically achievable in this relatively short time period. Factors that might influence the achievement of indirect supervision levels include the length of the clerkship rotation and the UME curriculum structure both before and during the clerkship. The division between the envisioned and actual entrustment-supervision level of graduating medical students has been described similarly in the context of pediatrics [40]. Longitudinal clerkships might provide advantages in this respect [12].

### Timing and frequencies of learner-initiated WBAs

Students predominantly used EPAs 1, 2 and 8 for WBAs. On the one hand, this might be a result of our specific curricular structure, with at least one WBA required for EPAs 1 and 2. On the other hand, students completed more WBAs than required and covered all other EPAs, except for EPA 9. Our interpretation of this pattern is that it most likely reflects those core EPAs that were most appropriate for the training level of early clinical students in the workplace and, thus, the activities that were predominantly entrusted to students. Other studies in UME on EPA progression and assessment have shown similar patterns, with students achieving higher supervision levels at an early phase for EPAs related to history taking, physical examination, presentation, and documentation [34, 49].

However, the observed pattern might also reflect the discrepancy between core EPAs designed for UME in general and specialty-specific clinical learning environments as described in other contexts [49]. There might be unique aspects to the practice and learning of clinical psychiatry [50] such as initially managing acutely suicidal or agitated patients (core EPA 6: Recognize & treat an emergency) that would make WBAs of certain (nested) EPAs especially important for achieving competency-based learning goals. Our findings support the need for the systematic development of EPAs for UME through a construct validity lens that takes into account the specific clinical context and informs clerkship curriculum design [39].

The observed distribution of WBAs also indicates that students used formative WBAs throughout their clerkship rotation without cherry-picking clinical situations at the end of the rotation. One potential reason for this might be the formative nature of our WBAs as opposed to graded WBAs, as has been described in the context of a comparable clerkship rotation program [43].

### Narrative feedback from the WBA tool and student evaluations

We found that, despite the time constraints of clinical staff and the challenges of WBAs as described in the literature [15, 30, 43], most WBA forms contained high-quality narrative feedback, which supports the findings from GME [25, 51]. We did not find any red-flag elements in the feedback narratives. This finding was also supported by external students’ evaluations of the novel WBA format, as well as a trend towards perceived higher benefits and more actionable feedback. Therefore, we conclude that using an entrustment-supervision scale with prompts for written feedback facilitates targeted feedback. A recent review has revealed that WBAs with entrustment-supervision scales and narrative feedback might be under-utilized in the context of EPAs [2].

In addition, we found that students approached different health professionals, including psychologists and nurses, to solicit feedback on clinical activities. This suggests more systematic exploration of the potential for an inter-professional educational design of clerkship curricula should be conducted. We are unaware of any studies on formalized inter-professional entrustment processes in clerkships. The narratives also addressed different CanMEDS roles. Given the workplace-based portfolios in clerkships, our data indicate that narrative feedback from WBAs with entrustability-supervision scales might be a valuable source of information for the Medical Expert role, which has been less represented (in comparison to the Professional and Communicator roles) in learning portfolios that were examined in a multi-center study [52]. A better understanding of the perceptions of students and residents with regard to the feedback process based on narratives is necessary to identify the reasons why a fifth of WBA forms did not contain any narratives [18].

### Limitations

Due to the observational design of our study, we cannot make any causal inferences about the relationship between WBAs based on entrustment-supervision scales and changes in the perceived or actual need for supervision. Furthermore, the context of psychiatry as a clinical specialty might have influenced the relative focus on clinical interviewing and communication skills for the WBAs. However, we collected data longitudinally, with both quantitative and qualitative methods, using a representative sample of the clerkship cohort. This allowed us to gain a multidimensional perspective on WBAs with a prospective entrustment-supervision scale and their formative value based on changes in self-entrustment ratings and resultant narrative feedback content and quality.

## Conclusions

Using a WBA tool with an entrustment-supervision scale and prompts for written narratives appeared to facilitate targeted feedback. WBA use was correlated with students’ development towards self-entrusted, indirect supervision levels that are a prerequisite to achieving competency-based learning goals. This WBA format should, therefore, be considered for inclusion in core clerkships to support self-regulated learning. Factors influencing the achievement of indirect supervision levels, and how to leverage inter-professional clinical supervision, need further exploration.

## Supplementary Information

**Additional file 1: Figure S1.** Translated WBA-tool based on a prospective entrustment-supervision scale.

## Data Availability

Anonymized quantitative data available on request.

## Abbreviations

EPA: Entrustable professional activity
UME: Undergraduate medical education
GME: Graduate medical education
WBA: Workplace-based assessment
