# Supplementary material for: Workplace-based assessments of entrustable professional activities in a psychiatry core clerkship: an observational study
Source: BMC Med Educ. 2021 Apr 21;21:223. doi: 10.1186/s12909-021-02637-4 (PMC8059233; doi:10.1186/s12909-021-02637-4)
Supplement: Supplementary file 1 — Additional file 1: Figure S1. Translated WBA-tool based on a prospective entrustment-supervision scale. [file 12909_2021_2637_MOESM1_ESM.docx]

**Figure S1.** Translated WBA-tool based on a prospective entrustment-supervision scale

**Core clerkship rotation (Psychiatry)**

**Sample workplace-based assessment (WBA) form**

**Date: __________ Ward: _________________**

**Starting situation** (age / gender / legal status / referring physician / (Diagnosis, e.g.; 36-year-old male patient, voluntary hospitalization, referred from general practitioner with major depressive episode).

.......................................................................................................................................................

At least four assessment ratings per clerkship rotation, which must include EPA 1 and EPA 2.

| **Activity** (**EPAs** =Entrustable Professional Activities) | **Rating:** |
| --- | --- |
| ☐ Take a patient’s psychiatric history **(EPA 1)** |  |
| ☐ Assess physical & mental status **(EPA 2)** |  |
| ☐ Prioritize a psychiatric differential diagnosis **(EPA 3)** |  |
| ☐ Order & interpret tests for a psychiatric patient (**EPA 4)** |  |
| ☐ Perform general procedures (e.g., initiate involuntary treatment) **(EPA 5)** |  |
| ☐ Recognize & treat psychiatric emergencies **(EPA 6)** |  |
| ☐ Prescribe & develop a management plan for a psychiatric patient **(EPA 7)** |  |
| ☐ Document & present a clinical encounter with a psychiatric patient **(EPA 8)** |  |
| ☐ Identify & report opportunities to improve patient safety in a psychiatric hospital **(EPA 9)** |  |
| ☐ ___________________________________________(Description of activity) |  |

**Supervision scale: 1 (observation only) - 6 (indirect supervision)**

I (clinical supervisor) think the student will need the following level of supervision for the observed activity:

1 = Observe only

2 = The student can do this as a co-activity with the supervisor

3 = The student can do this activity if the supervisor is present

4 = The student can do this if the supervisor completely repeats the activity
5 = The student can do this if the supervisor repeats the important parts of the activity

6 = The student can do this if he/she can ask for help when needed (indirect supervision)

**Specific feedback** (What was done well? What can be improved? Next steps?):

.......................................................................................................................................................

.......................................................................................................................................................

.......................................................................................................................................................

I (student) confirm, that I have been observed by my clinical supervisor for the marked activities and that I received specific feedback.

| Clinical supervisor (professional role): | Student: |
| --- | --- |
| Signature: | Signature: |

**Note: The filled and signed forms must be presented to the clerkship director at the end of the clerkship rotation. The original form belongs to the student.**
